# Is the long-term mortality similar in COVID-19 and community-acquired pneumonia?

**DOI:** 10.3389/fmed.2023.1236142

**Published:** 2023-10-10

**Authors:** Raúl Méndez, Paula González-Jiménez, Ana Latorre, Noé Mengot, Rafael Zalacain, Luis A. Ruiz, Leyre Serrano, Pedro P. España, Ane Uranga, Catia Cillóniz, David Hervás, Antoni Torres, Rosario Menéndez, NEUMONAC, RECOVID, Pedro Pablo España, Luis Borderías, Olga Rajas, Jordi Almirall, Rafael Zalacaín, Montserrat Vendrell, Salvador Bello, Isabel Mir, Concepción Morales, Luis Molinos, Ricard Ferrer, MªLuisa Briones, Rosa Malo, Itxaso Sayago Reza, Wanda Almonte Batista, Laura Moreno Galarraga, Oriol Sibila Vidal, Juan Luis Rodríguez Hermosa, Gianna Vargas Centanaro, Blanca de Vega Sánchez, Eduardo Solís García, Ester Rodríguez Florez, María José, Chourio Estaba, María Molina Molina, Jaume Bordas, María Estela González Castro, Diana Badenes Bonet, Marisol Domínguez Álvarez, Eli N. Pérez-Rodas, Alejandra Marín Arguedas, Berta Román Bernal, Graciliano Estrada Trigueros, Selene Cuenca Peris, Margarita Martín Royo, Miguel Torres García, José Portillo Sánchez, Francisca Lerenas Bernal, María Salome Ros Braquehais, José Alfonso García Guerra, María Dolores Martínez Pitarch, Iván Arroyo Fernández, Virginia Guevara Velázquez, Pilar Martínez Olondris, Marco Francisco Pereyra Barrionuevo, Javier Lázaro Sierra, Paloma Clavería, Aurelio Luis Wangüemert Pérez, José Joel Ruiz Lacambra, Noelia Fernández Ramos, Sara Guanche Dorta, Abigail Macias Paredes, David de la Rosa Carrillo, Esther Palones Femenia, Inés Podzamczer Valls, Patricia Peñacoba Toribio, Pilar Muñoz Zara, Rocío García García, María del Mar Marrube Fernández, Laura Villar Aguilar, Santiago de Jorge Domínguez Pazos,, Tara Pereiro Brea, Ana Pando-Sandoval, Marta María García Clemente, Amelia Alzueta Álvarez, Estela García Coya, Elizabeth de Freitas González, Pedro Pablo España Yandiola, Ane Uranga, Beatriz Raboso Moreno, Carolina Panadero, Araceli Abad, Irene Cano, Iria Pérez Orbis, Carolina Gotera Rivera, Celia Ruiz Pérez, Rosario Menéndez Villanueva, Raúl Méndez, Ana Latorre, Paula González, Teresa Ramírez Prieto, Miguel Ángel Salvador Maya, Claudia Valenzuela, José M. Cifrián Martínez, Juan Marco Figueira Gonçalves, Adrián Baeza Ruiz, Andrea Expósito Marrero, Nikita Gurbani, Rosa Malo de Molina Ruiz, Marina Galdeano Lozano, Manuel Villanueva Montes, Nuria Toledo Pons, Luisa Ramón Clar, Alba Esperanza Barrios, Pilar Cejudo Ramos, Cecilia López Ramírez, Mirella Gaboli, Virginia Almadana Pacheco, Ferran Eduard Barbé Illa, Anna Clara, Nuria Gutiérrez González, Eva Cabrera César

**Affiliations:** ^1^Pneumology Department, La Fe University and Polytechnic Hospital, Valencia, Spain; ^2^Respiratory Infections, Health Research Institute La Fe (IISLAFE), Valencia, Spain; ^3^Centro de Investigación Biomédica en Red de Enfermedades Respiratorias (CIBERES), Instituto de Salud Carlos III, Madrid, Spain; ^4^Department of Medicine, University of Valencia, Valencia, Spain; ^5^Pneumology Department, Cruces University Hospital, Barakaldo, Spain; ^6^Department of Immunology, Microbiology and Parasitology, Facultad de Medicina y Enfermería, Universidad del País Vasco/Euskal Herriko Unibertsitatea UPV/EHU, Leioa, Spain; ^7^Pneumology Department, Galdakao-Usansolo Hospital, Galdacano, Spain; ^8^Department of Medicine, University of Barcelona, Barcelona, Spain; ^9^Faculty of Health Sciences, Continental University, Huancayo, Peru; ^10^August Pi i Sunyer Biomedical Research Institute (IDIBAPS), Barcelona, Spain; ^11^Data Science, Biostatistics and Bioinformatics, Health Research Institute La Fe (IISLAFE), Valencia, Spain; ^12^Department of Applied Statistics and Operational Research and Quality, Universitat Politècnica de València, Valencia, Spain; ^13^Pneumology Department, Hospital Clinic of Barcelona, Barcelona, Spain

**Keywords:** long-term, mortality, COVID-19, pneumonia, Bayesian

## Abstract

**Introduction:**

There are no data on the association of type of pneumonia and long-term mortality by the type of pneumonia (COVID-19 or community-acquired pneumonia [CAP]) on long-term mortality after an adjustment for potential confounding variables. We aimed to assess the type of pneumonia and risk factors for long-term mortality in patients who were hospitalized in conventional ward and later discharged.

**Methods:**

Retrospective analysis of two prospective and multicentre cohorts of hospitalized patients with COVID-19 and CAP. The main outcome under study was 1-year mortality in hospitalized patients in conventional ward and later discharged. We adjusted a Bayesian logistic regression model to assess associations between the type of pneumonia and 1-year mortality controlling for confounders.

**Results:**

The study included a total of 1,693 and 2,374 discharged patients in the COVID-19 and CAP cohorts, respectively. Of these, 1,525 (90.1%) and 2,249 (95%) patients underwent analysis. Until 1-year follow-up, 69 (4.5%) and 148 (6.6%) patients from the COVID-19 and CAP cohorts, respectively, died (*p* = 0.008). However, the Bayesian model showed a low probability of effect (PE) of finding relevant differences in long-term mortality between CAP and COVID-19 (odds ratio 1.127, 95% credibility interval 0.862–1.591; PE = 0.774).

**Conclusion:**

COVID-19 and CAP have similar long-term mortality after adjusting for potential confounders.

## Introduction

COVID-19 has shown a capacity to cause devastating outcomes. Fortunately, the emergence of effective vaccines and treatments have significantly mitigated this effect (1). However, there remains a concern about long-term mortality and other health consequences. Previous studies have demonstrated an increase in long-term mortality in patients with community-acquired pneumonia (CAP) compared to the general population. In those with CAP, approximately 9% of survivors with an acute episode who have required hospitalization die within a year (2, 3). Contrarily, such mortality in patients COVID-19 has been reported as very low (4). To our knowledge, though, there are no data comparing the association of type of pneumonia (COVID-19 or CAP) with long-term mortality after an adjustment for potential confounding variables. Therefore, our aim was to assess the contribution of potentially confounding risk factors to the long-term odds of death in COVID-19 versus CAP.

## Methods

We designed a retrospective analysis of two large, prospective and multicentre cohorts of hospitalized patients with COVID-19 and CAP. Patients were follow-up for a year after discharge. The COVID-19 cohort comprised patients from the RECOVID study and included patients admitted during 2020. Briefly, RECOVID is a registry of 49 Spanish hospitals with hospitalized patients with confirmed SARS-CoV-2 infection by reverse transcription polymerase chain reaction and with new infiltrates on chest X-ray or CT scan. The study received approval by the local ethics committees (UIC-IBU-2020-03). Furthermore, the CAP cohort included hospitalized patients from 15 Spanish hospitals between January 2012 and August 2015 (NEUMONAC study). For this cohort, patients met inclusion criteria if they received a diagnosis for pneumonia based on a new radiologic infiltrate and the presence of at least two compatible clinical symptoms. Exclusion criteria for both cohorts included admission within the previous 15 days and immunosuppression. We also excluded all those admitted to the intensive care unit (ICU; 13.5% of the total cohort) due to the high variability of ICU admission criteria during the pandemic. The study received approval by the ethics committee of each hospital, and patients signed an informed consent (2013/0204).

The main outcome under study was 1-year mortality in patients who were hospitalized in conventional ward and later discharged. We compared the association of type of pneumonia with long-term mortality after adjusting for variables selected by clinical decisions and per previous literature. Those variables were age, sex, nursing-home residency, smoking history, Charlson Comorbidity Index (CCI), bilateral radiologic involvement, CURB65 and vascular complications during hospitalization (3). Cardiovascular complications included acute coronary syndrome, heart failure, arrhythmia, cerebrovascular accident and venous thromboembolic disease (5). We summarized data as *n* (%) or median (interquartile range). Furthermore, controlling for all previously mentioned covariates, we adjusted a Bayesian logistic regression model to assess associations between the type of pneumonia type and 1-year mortality. The main reason for using Bayesian logistic regression was to accommodate the multiple imputation procedure in a natural and straightforward way. Using Bayesian inference provides more robust estimates of the model parameters and also captures the uncertainty of the different parameters in a more reliable way (6). A multiple imputation procedure was implemented with missing data to improve the robustness of our results. Briefly, we created 50 different imputed datasets using the Multivariate Imputation by Chained Equations (MICE) approach (7). Then, a Bayesian logistic regression model was fitted on each of the varying imputed datasets; all models were combined to get the pooled estimates. Flat prior distributions (non-informative) for each of the coefficients were used. We computed 95% credibility intervals for all the estimates and estimated the probability of effect (MPE) following the procedure of Makowski et al. (8). Credibility intervals have a probabilistic interpretation regarding the parameter’s possible values (i.e., the area under the posterior distribution’s curve between two points is the probability that the value of the parameters lies between these two points). Statistical analyzes were performed using R (version 4.2.2) and R packages clickR (version 0.9.27), MICE (version 3.15.0) and BRMS (version 2.18.0).

## Results

**Figure 1:**
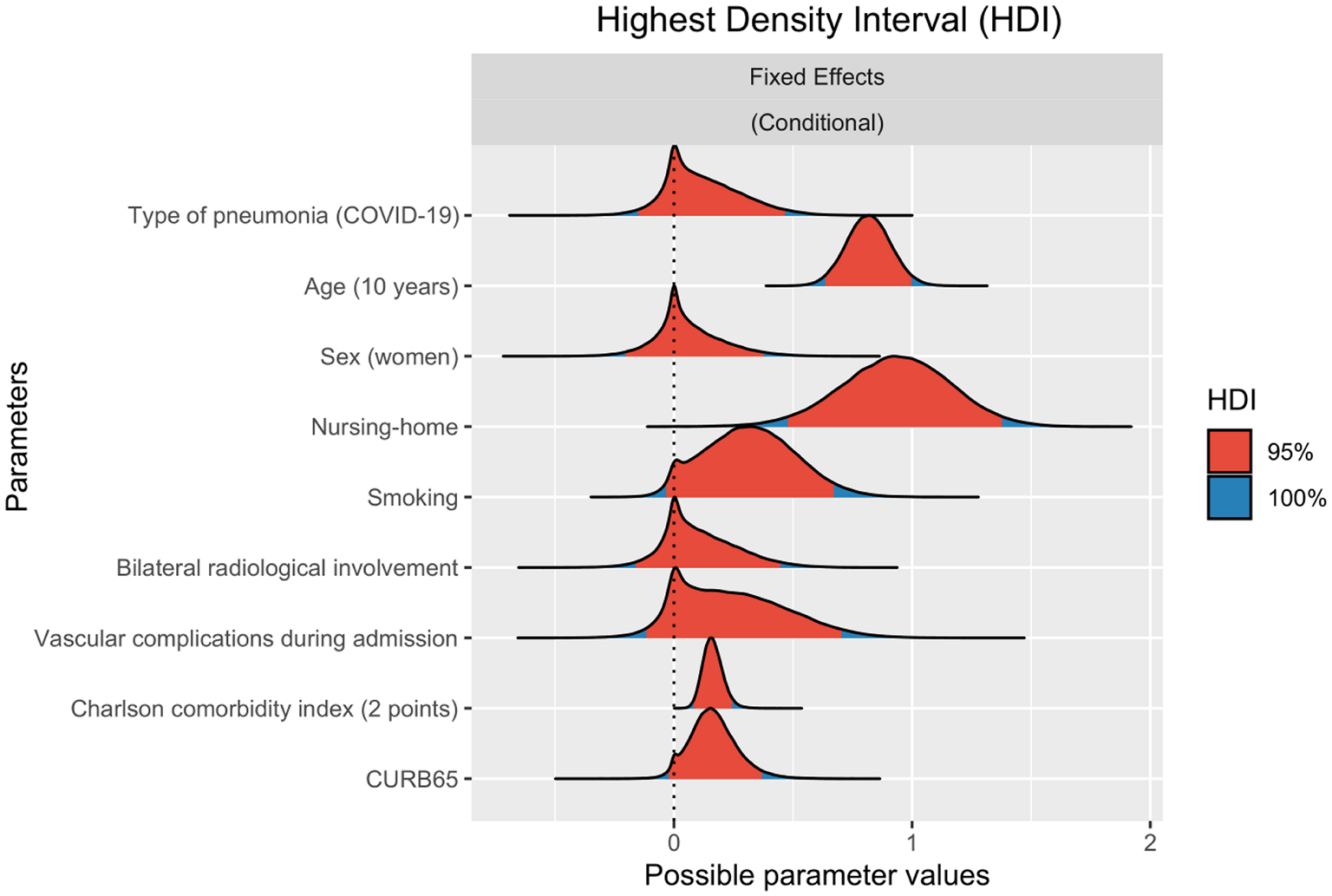
Approximate probability distributions for estimates of different logistic regression model parameters for 1-year mortality. Odds ratio are shown in natural log scale. 95 and 100% highest density intervals (HDI) for each estimate are colored in red and blue, respectively.

We included a total of 1,693 and 2,374 discharged patients in the COVID-19 and CAP cohorts, respectively. Of these, 1,525 (90.1%) and 2,249 (95%) patients underwent analysis after we excluded those with missing data on long-term mortality. Briefly, patients with CAP were older (71 [56–80] vs. 63 [52–73]), with the highest CCI score (1 [0–3] vs. 0 [0–1]) and a greater initial severity measured by CURB65 (1 [1–2] vs. 1 [0–1]) when compared to patients with COVID-19 (Table 1). In contrast, there were fewer smokers (former or current) in the COVID-19 cohort (35.3% vs. 55.7%). As expected, more bilateral radiological involvement was found in COVID-19 (67.6% vs. 13.9%). We found similar data per female sex (39.7% vs. 43.1%), nursing-home residency (4.4% vs. 4.6%) and vascular complications during admission (6.4% vs. 4%) in both cohorts (CAP vs. COVID-19). In 201 cases of the CAP cohort, the etiology was viral. Since discharge until 1-year follow-up, 69 (4.5%) and 148 (6.6%) patients from the COVID-19 and CAP cohorts, respectively, died (*p* = 0.008). After being adjusted for the selected variables, the Bayesian model showed a low probability of effect (PE) of finding relevant differences in long-term mortality between CAP and COVID-19 (odds ratio [OR] 1.127, 95% credibility interval [CI] 0.862–1.591; PE = 0.774; Figure 1). A very high PE in long-term mortality was found for age (10-year intervals; OR 2.264, 1.891–2.717; PE > 0.999), nursing-home residency (OR 2.54, 1.602–3.94; PE = 0.999), CCI (2-points intervals; OR 1.177, 1.094–1.288; PE > 0.999), a history of smoking (OR 1.373, 0.987–2.019; PE = 0.962) and CURB65 (OR 1.179, 0.99–1.476; PE = 0.961). In addition, vascular complications (OR 1.272, 0.901–2.062; PE = 0.866) also showed a high probability of conferring a negative effect on mortality. Furthermore, sex (OR 1.06, 0.822–1.467; PE = 0.649) and bilateral radiologic involvement (OR 1.113, 0.86–1.577; PE = 0.752) had a low probability of being relevant as it concerns death at 1-year follow-up. We observed similar results without an imputation procedure (data not shown).

**Table 1:** Characteristics for CAP and COVID-19 cohorts.

	CAP		COVID-19	
	*N*	2,249	*N*	1,525
Age	2,249	71 (56–80)	1,525	63 (52–73)
Sex (female)	2,249	893 (39.7)	1,525	657 (43.1)
Smoking (former or current)	2,123	1,182 (55.7)	1,481	523 (35.3)
Nursing-home	2,243	99 (4.4)	1,483	68 (4.6)
Diabetes	2,246	524 (23.3)	1,525	253 (16.6)
Heart disease	2,247	653 (29.1)	1,525	188 (12.3)
Renal disease	2,242	225 (10)	1,525	72 (4.7)
COPD	2,218	408 (18.4)	1,525	100 (6.6)
CCI	1867	1 (0–3)	1,525	0 (0–1)
CURB65	2,231	1 (1–2)	1,276	1 (0–1)
Bilateral radiological involvement	2,249	313 (13.9)	1,390	940 (67.6)
Vascular complications	2,243	144 (6.4)	1,295	52 (4)
1-year mortality after discharge	2,249	148 (6.6%)	1,525	69 (4.5%)

Data are shown as *n* (%) or median (interquartile range). CCI, Charlson Comorbidity Index; COPD, chronic obstructive pulmonary disease.

## Discussion

Age, nursing-home residency, smoking history and comorbidity are recognized factors related to long-term mortality (3). We found that the odds of death doubled for each 10-year interval in age or nursing-home residency. Secondly, as has been previously shown, the greater the CCI and CURB65 scores, the greater the mortality (9). Vascular events, especially in vulnerable patients, will leave sequelae that condition higher mortality. We showed that vascular complications are highly probable in conferring a negative effect on 1-year mortality. CAP severity is known to cause cardiovascular complications, which can worsen long-term prognosis (10, 11). The COVID-19 cohort showed lower mortality, perhaps due to a younger age, than the CAP cohort. However, in the analysis, there were no relevant differences in mortality across both cohorts. This finding highlights long-term impact, not only in morbidity or long COVID.

Our study has several strengths and limitations. This is the first study to compare long-term mortality in individuals with COVID-19 or CAP Here we show that, similar to CAP, COVID-19 has considerable long-term mortality that has not been adequately addressed. Nevertheless, recruitment of these cohorts took place in different periods so we cannot rule out its possible effect on the results. Second, we used the CURB65 score, which has not been completely tested in COVID-19 (12, 13).

In conclusion, COVID-19 and CAP have similar long-term mortality after we adjusted for potential confounders. Efforts to treat COVID-19 must also focus on the long-term consequences and not just on the acute phase.

## Data availability statement

The raw data supporting the conclusions of this article will be made available by the authors, without undue reservation.

## Ethics statement

The studies involving humans were approved by CEIM - Hospital Universitario y Politécnico La Fe. The studies were conducted in accordance with the local legislation and institutional requirements. The participants provided their written informed consent to participate in this study.

## Author contributions

RaM and RoM studied the design, drafted the manuscript, and act as the guarantors. RaM, PG-J, AL, NM, RZ, LR, LS, PE, AU, CC, and AT involved in the patient enrolment. DH done the statistical analysis. All authors contributed to the article and approved the submitted version.

## List of NEUMONAC investigators

Pedro Pablo España (Hospital de Galdakao, Galdakao); Luis Borderías (Hospital San Jorge, Huesca); Olga Rajas (Hospital La Princesa, Madrid); Jordi Almirall (Hospital de Mataró, Mataró); Rafael Zalacaín (Hospital de Cruces, Bilbao); Montserrat Vendrell (Hospital Josep Trueta, Girona); Salvador Bello (Hospital Miguel Servet, Zaragoza); Isabel Mir (Hospital Son Llàtzer, Palma de Mallorca); Concepción Morales (Hospital Virgen de las Nieves, Granada); Luis Molinos (Hospital Universitario Central de Asturias, Oviedo); Ricard Ferrer (Hospital Mutua Terrasa, Terrasa); Mª Luisa Briones (Hospital Clínico Universitario, Valencia); Rosa Malo (Hospital Puerta de Hierro, Majadahonda).

## List of RECOVID investigators

Itxaso Sayago Reza (Clínica Asunción); Wanda Almonte Batista (Complejo Hospitalario Univ. de Albacete); Laura Moreno Galarraga (Complejo Hospitalario Univ. de Navarra); Oriol Sibila Vidal (Hospital Clínic); Juan Luis Rodríguez Hermosa and Gianna Vargas Centanaro (Hospital Clínico San Carlos); Blanca de Vega Sánchez, Eduardo Solís García, Ester Rodríguez Florez, and María José Chourio Estaba (Hospital Clínico Univ. de Valladolid); María Molina Molina and Jaume Bordas (Hospital de Bellvitge); María Estela González Castro (Hospital de Torrecárdenas); Diana Badenes Bonet and Marisol Domínguez Álvarez (Hospital del Mar); Eli N. Pérez-Rodas and Alejandra Marín Arguedas (Hospital Dos de Maig); Berta Román Bernal (Hospital Dr. José Molina Orosa); Graciliano Estrada Trigueros (Hospital General de Segovia); Selene Cuenca Peris, Margarita Martín Royo, and Miguel Torres García (Hospital Gral. Univ. de Castellón); José Portillo Sánchez (Hospital Gral. Univ. de Ciudad Real); Francisca Lerenas Bernal and María Salome Ros Braquehais (Hospital Gral. Univ. Santa Lucía); José Alfonso García Guerra (Hospital La Mancha Centro); María Dolores Martínez Pitarch and Iván Arroyo Fernández (Hospital Lluis Alcanyís); Virginia Guevara Velázquez (Hospital Ntra. Sra. de Sonsoles); Pilar Martínez Olondris (Hospital Plató); Marco Francisco Pereyra Barrionuevo (Hospital público de Monforte de Lemos); Javier Lázaro Sierra and Paloma Clavería (Hospital Royo Villanova); Aurelio Luis Wangüemert Pérez, José Joel Ruiz Lacambra, Noelia Fernández Ramos, and Sara Guanche Dorta (Hospital San Juan de Dios de Tenerife); Abigail Macias Paredes (Hospital Sant Jaume de Calella); David de la Rosa Carrillo, Esther Palones Femenia, Inés Podzamczer Valls, and Patricia Peñacoba Toribio (Hospital Santa Creu i Sant Pau); Pilar Muñoz Zara (Hospital de Jérez); Rocío García García (Hospital Univ. 12 de Octubre); María del Mar Marrube Fernández, Laura Villar Aguilar, Santiago de Jorge Domínguez Pazos, and Tara Pereiro Brea (Hospital Univ. A Coruña); Ana Pando-Sandoval and Marta María García Clemente (Hospital Univ. Central de Asturias); Amelia Alzueta Álvarez, Estela García Coya, and Elizabeth de Freitas González (Hospital Univ. de Cabueñes); Pedro Pablo España Yandiola and Ane Uranga (Hospital Univ. de Galdakao-Usansolo); Beatriz Raboso Moreno, Carolina Panadero, Araceli Abad, and Irene Cano (Hospital Univ. de Getafe); Iria Pérez Orbis (Hospital Univ. Fundación Alcorcón); Carolina Gotera Rivera and Celia Ruiz Pérez (Hospital Univ. Fundación Jiménez Díaz); Rosario Menéndez Villanueva, Raúl Méndez, Ana Latorre, and Paula González (Hospital Univ. i Politècnic La Fe); Teresa Ramírez Prieto and Miguel Ángel Salvador Maya (Hospital Univ. Infanta Sofía); Claudia Valenzuela (Hospital Univ. La Princesa); José M. Cifrián Martínez (Hospital Univ. Marqués de Valdecilla); Juan Marco Figueira Gonçalves, Adrián Baeza Ruiz, Andrea Expósito Marrero, and Nikita Gurbani (Hospital Univ. Ntra. Sra. Candelaria). Rosa Malo de Molina Ruiz (Hospital Univ. Puerta de Hierro); Marina Galdeano Lozano (Hospital Univ. Sagrat Cor); Manuel Villanueva Montes (Hospital Univ. San Agustín). Nuria Toledo Pons and Luisa Ramón Clar (Hospital Univ. Son Espases); Alba Esperanza Barrios (Hospital Univ. Torrejón); Pilar Cejudo Ramos, Cecilia López Ramírez, and Mirella Gaboli (Hospital Univ. Virgen del Rocío); Virginia Almadana Pacheco (Hospital Univ. Virgen Macarena); Ferran Eduard Barbé Illa and Anna Clara (Hospital Univ. Arnau de Vilanova); Nuria Gutiérrez González (Hospital Virgen de la Luz); Eva Cabrera César (Hospital Virgen de la Victoria).
